# Molecular Genetic Analysis of the Autosomal Recessive Non-Syndromic Inherited Retinitis Pigmentosa

**DOI:** 10.7759/cureus.37933

**Published:** 2023-04-21

**Authors:** Faiza Habib, Muhammad Yasin, Namal ., Shaheryar ., Areej Nasir, Abrar Hussain, Chinonso Ndubuisi, Hiba Azam, Muhammad Sajid, Arsalan Rasheed

**Affiliations:** 1 Department of Molecular Biology and Genetics, Institute of Basic Medical Sciences, Khyber Medical University, Peshawar, PAK; 2 Department of Biotechnology and Genetic Engineering, Kohat University of Science and Technology, Kohat, PAK; 3 Internal Medicine, United Medical and Dental College, Faisalabad, PAK; 4 Medicine, University College of Medicine and Dentistry, Lahore, PAK; 5 Medicine, Quaid-e-Azam Medical College, Bahawalpur, PAK; 6 Department of Biological Sciences, International Islamic University, Islamabad, PAK; 7 Family Medicine, Humboldt Park Health, Chicago, USA; 8 Surgery, University College of Medicine and Dentistry, Lahore, PAK; 9 Department of Biotechnology and Genetic Engineering, International Islamic University, Islamabad, PAK; 10 Department of Zoology, Faculty of Life and Chemical Sciences, Abdul Wali Khan University Mardan, Mardan, PAK

**Keywords:** retinitis pigmentosa 1 (rp1), crumbs homolog 1 (crb1), pakistan, retinitis pigmentosa, visual impairment, linkage analysis, genetic mutation

## Abstract

Introduction: 90% of visually impaired people live in developing countries. There are various types of vision impairment, but the focus of the current study is retinitis pigmentosa (RP). Up to now, 150 mutations have been reported that are linked with RP.

Methodology: Healthy and affected members from two Pakistani families (RP01 and RP02) segregating autosomal recessive RP were selected for DNA extraction. PCR was conducted, and the amplified PCR products were analyzed using Polyacrylamide Gel Electrophoresis (PAGE) and visualized in the Gel Doc system for linkage analysis. The Gene Hunter 2.1r5 tool in the Simple Linkage v5.052 beta software suite was used to conduct multipoint parametric linkage analysis on the two consanguineous families examined on the 6K Illumina array. Exons and intron-exon borders of all known arRP genes found in homozygous areas were sequenced in the matching probands using a 3130 automated sequencer and the Big Dye Terminator Cycle Sequencing Kit v3.1. The mutation study was carried out using the AlaMut 1.5 program.

Results: In both families, linkage analysis was performed using microsatellite marker DIS422 for gene *crumbs homolog 1 (CRB1)* and microsatellite marker D8S2332 for gene *Retinitis Pigmentosa 1 (RP1)*. Multipoint linkage analysis identifies genomic regions that could potentially contain the genetic defect. In family RP01, only a single peak with a maximal multipoint LOD score of 3.00 was identified on chromosome 1, whereas in family RP02, multiple peaks with multipoint LOD scores of 1.80 were identified on chromosome 8. Analysis of the *CRB1* gene revealed a homozygous substitution of glycine for valine (c.1152T>G; p.V243G), whereas the *RP1* gene demonstrated that leucine was substituted for proline as a result of cytosine to thymine transfer (c.3419C>T; p. P1035L).

Conclusion: Homozygosity mapping is a powerful method for finding genetic abnormalities that are both precise and comprehensive for identifying harmful variations in consanguineous families. This method is invaluable for providing accurate clinical diagnostic and genetic advice in remote regions of Pakistan while also increasing knowledge about autosomal recessive diseases and the dangers of mixing.

## Introduction

Dr. Donders coined the term “retinitis pigmentosa” (RP) in 1857 [1,2], which identifies a group of hereditary retinal illnesses characterized by a steady decline of rods and cones (light-sensing cells) [2,3], which can result in severe vision impairment or blindness [4,5]. RP affects 1 in every 4000 people in the United States and approximately 1 in 5000 people worldwide [6], making it one of the most common genetic causes of vision impairment worldwide. RP is heterogeneous clinically and genetically and is categorized into non-syndromic, syndromic, and systemic [7,8].

The inheritance patterns of non-syndromic RP may be autosomal recessive, which is 50%-60% (the most prevalently inherited RP worldwide), an autosomal dominant pattern in about 30%-40% of cases, or X-linked patterns, which are almost 5%-15% prevalent [7]. Sporadic cases of the mitochondrial or digenic mode of inheritance are also reported [9]. Usher syndrome (the most prevalent cause of collective blindness and deafness) and Bardet-Biedl syndrome (in this condition, RP is accompanied by polydactyly, obesity, renal abnormalities, hypogenitalism, and mental retardation) are the most common forms of syndromic RP [9,10].

More than 50 known genes cause retinitis pigmentosa, which constitutes only 50% of the prevailing disorder, while 22 genes are associated with autosomal dominant RP (adRP), 40 genes are associated with autosomal recessive RP (arRP), and five genes are associated with X-linked RP [5,7-9]. This research was carried out to identify the gene mutation that causes RP in Pakistani families using homozygosity mapping.

## Materials and methods

Ethical statement

Research approval was granted by the Research Ethical Committee of the International Islamic University, Islamabad, Pakistan, under the No. IIUI/BIOTECH/ASRB2016. All participants, including those who were impacted, their parents, and other regular family members of the afflicted households, provided written consent.

Genes selected in this research study

Researchers suggested numerous candidate genes associated with RP [3-6,8], but due to limited resources, only five genes were selected for the current study: 1) CRB1 (OMIM # 604210; RefSeq, Apr 2012), 2) RP1 (OMIM # 603937; RefSeq, Sep 2010), 3) TULP1 (OMIM # 602280; RefSeq, Nov 2016), 4) RPE65 (OMIM # 180069; RefSeq, Oct 2017), and 5) ABCA4 (OMIM # 601691; RefSeq, Jul 2008).

Selection of families

Two families, each having at least two affected individuals suffering from non-syndromic autosomal recessive RP, were selected for this study in KPK, Pakistan. Medical history and records were documented. Ophthalmologists of the local hospital examined the clinical characterization of affected and non-affected members of selected families, and disease-associated signs and symptoms were recorded (Tables 1, 2). The mode of inheritance was determined by pedigree analysis. 

**Table 1:** Clinical Examination of RP01 Family

Clinical Features	IV:1	IV:2	V:1	V:2	V:3
Sex	Male	Female	Female	Female	Male
Phenotype	Normal	Normal	Affected	Affected	Normal
Age	50 years	45 years	25 years	23 years	20 years
Speech Disorders	-	-	-	-	-
Deafness	-	-	-	-	-
Growth	Normal	Normal	Normal	Normal	Normal
Schooling	+	-	-	-	+
Night Blindness	-	-	-	-	-
Eyebrows	+	+	+	+	+
Lacrimal Gland	No Tearing	No Tearing	No Tearing	No Tearing	No Tearing
Sclera	White	White	White	White	White
Iris	Yes	Yes	Yes	Yes	Yes
Pupils	Black	Black	Black	Black	Black
Blurry Vision	No	No	No	No	No

**Table 2:** Clinical Examination of RP02 Family M: Male; F: Female; N: Normal and A: Affected

Clinical Features	V:1	V:2	VI:1	VI:2	VI:3	VI:4	VI:5	VI:6	VII: 1	VII:2	VII:3	VII:4
Gender	F	M	F	F	M	M	F	M	F	F	M	M
Phenotype	N	N	N	A	A	N	N	N	A	N	A	N
Age	70	65	37	35	33	30	27	31	11	09	07	04
Speech Disorders	-	-	-	-	-	-	-	-	-	-	-	-
Deafness	-	-	-	-	-	-	-	-	-	-	-	-
Growth	N	N	N	N	N	N	N	N	N	N	N	N
Schooling	-	-	-	-	+	+	-	+	-	-	-	-
Night Blindness	-	-	-	-	-	-	-	-	-	-	-	-
Eye Brows	+	+	+	+	+	+	+	+	+	+	+	+
Lacrimal Gland	No Tearing	No Tearing	No Tearing	No Tearing	No Tearing	No Tearing	No Tearing	No Tearing	No Tearing	No Tearing	No Tearing	No Tearing
Sclera	White	White	White	White	White	White	White	White	White	White	White	White
Iris	Yes	Yes	Yes	White	White	Yes	Yes	Yes	White	Yes	White	Yes
Pupils	Black	Black	Black	Black	Black	Black	Black	Black	Black	Black	Black	Black
Blurry Vision	No	No	No	No	No	No	No	No	No	No	No	No

Sample collection

Five milliliters (5ml) of a peripheral blood sample from each individual was collected by sterile syringe, using the venipuncture technique, in an EDTA vacutainer. Collected samples were stored at 4° C in a laboratory refrigerator till the extraction of genomic DNA.

Linkage analysis and homozygosity mapping

The genomic DNA of participants was extracted from peripheral blood using an organic technique (phenol-chloroform), and the isolated DNA samples were examined using a 1% agarose gel, as reported in our previously published article [8]. Following that, PCR was conducted, and the amplified PCR products were analyzed using polyacrylamide gel electrophoresis (PAGE) and visualized in the Gel Doc system (BioRad, Italy) for linkage analysis. The Gene Hunter 2.1r5 tool in the Simple Linkage v5.052 beta software suite was used to conduct multipoint parametric linkage analysis on the two consanguineous families examined on the 6K Illumina array. Exons and intron-exon borders of all known arRP genes found in homozygous areas were sequenced in the matching probands using a 3130 automated sequencer and the Big Dye Terminator Cycle Sequencing Kit v3.1. The mutation study was carried out using the Alamut 1.5 program.

## Results

Family description and assessment

**Figure 1:**
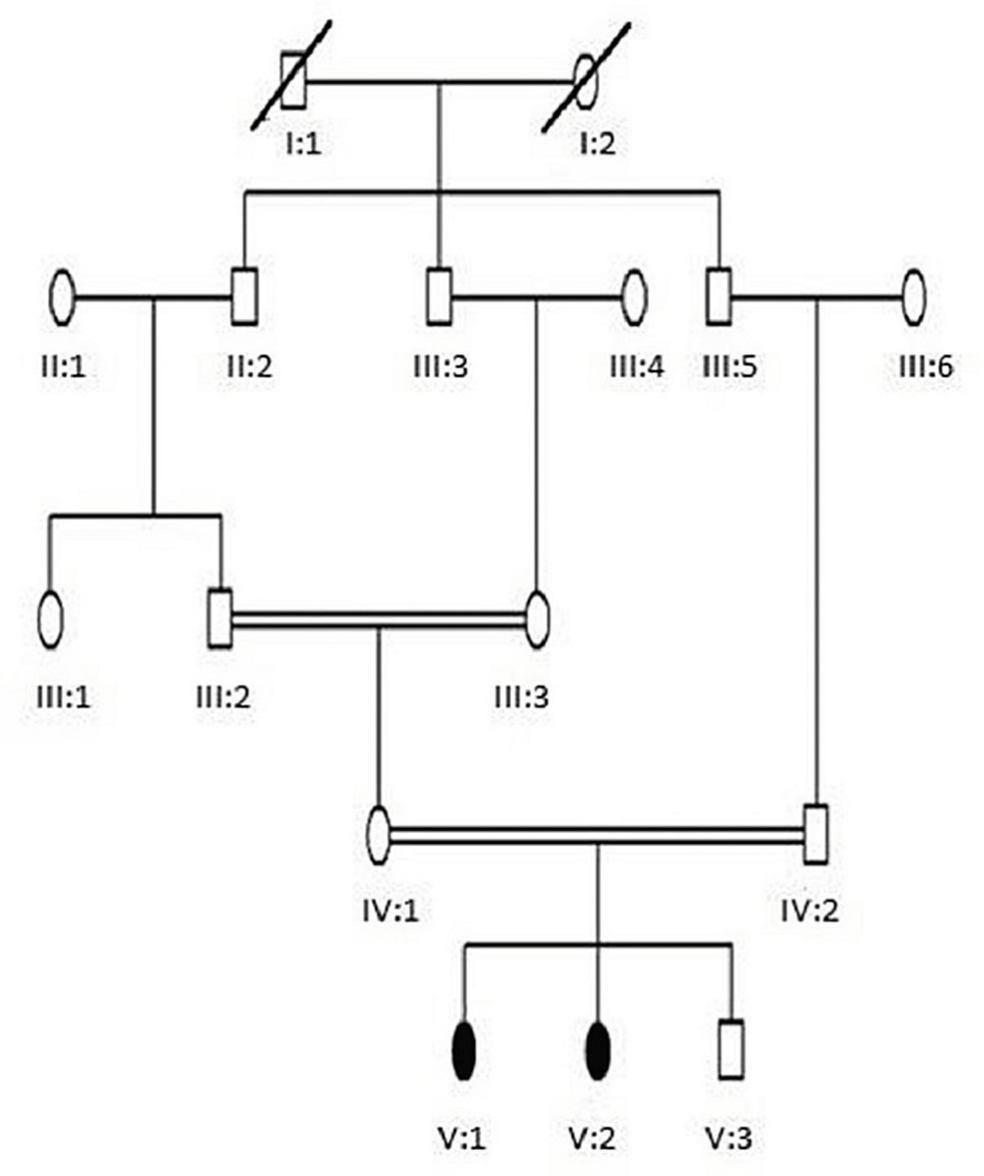
Family RP01 pedigree Squares show male members, while circles represent female individuals. Affected members of families were represented by filled symbols, while unfilled symbols showed non-affected members. A double line in pedigree represented consanguineous marriage. An oblique line on a square or circle showed deceased members. Roman numerals represented generations while Arabic numerals denoted individuals.

**Figure 2:**
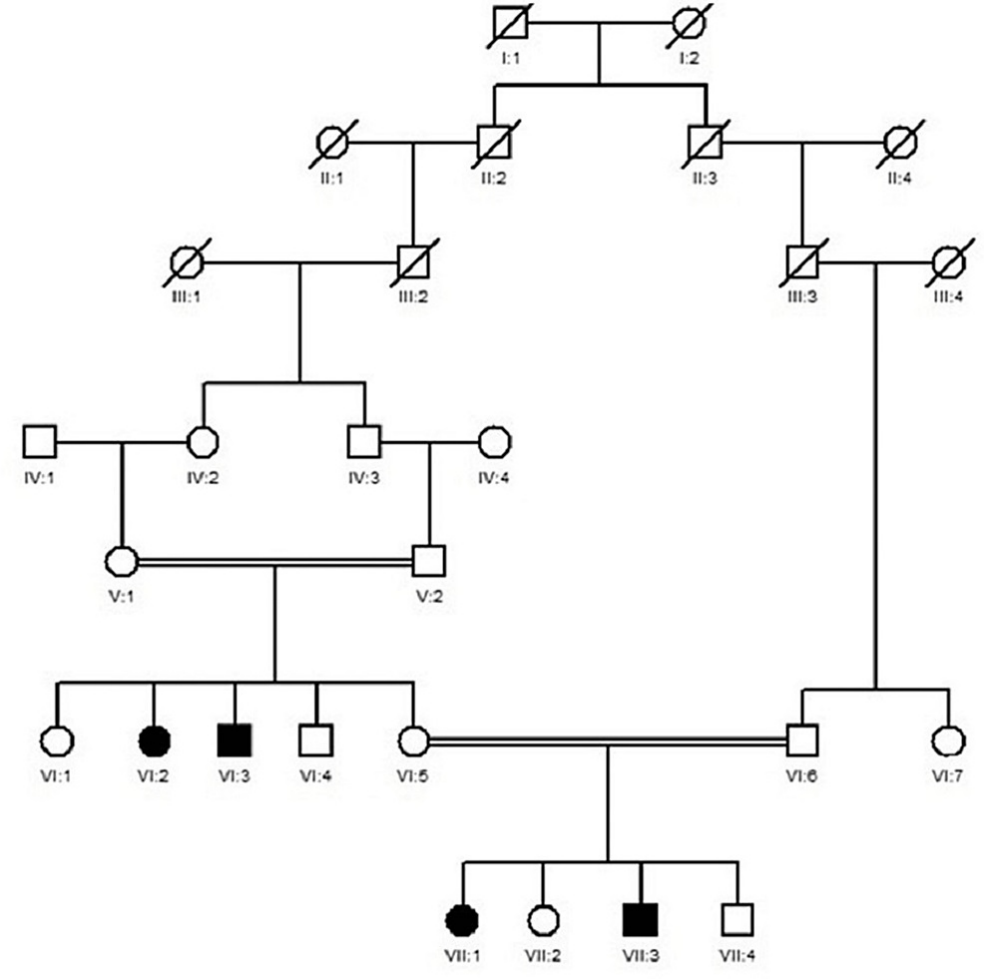
Family RP02 pedigree Squares show male members, while circles represent female individuals. Affected members of families were represented by filled symbols, while unfilled symbols showed non-affected members. A double line in pedigree represented consanguineous marriage. An oblique line on a square or circle showed deceased members. Roman numerals represented generations while Arabic numerals denoted individuals.

Current research includes two families of RP labeled as RP01 and RP02. The descriptions of both families are as follows: Family RP01 resides in the district of Bannu in the Khyber Pakhtunkhwa province of Pakistan. There are five generations in family RP01, with normal consanguineous parents (IV.1 and IV.2), two affected females (V:1 and V:2), and one normal male (V:3), with a pedigree showing an autosomal recessive pattern of inheritance (Figure 1). Family RP02 resides in the district of Shangla in Khyber Pakhtunkhwa, North of Pakistan. The family contains seven generations: non-affected first consanguineous parents (V.1 and V.2), two affected individuals, one affected female (VI.2) and one affected male (VI.3), and two normal females and one normal male with a pedigree showing an autosomal recessive pattern of inheritance (Figure 2).

Linkage analysis through homozygosity mapping

**Figure 3:**
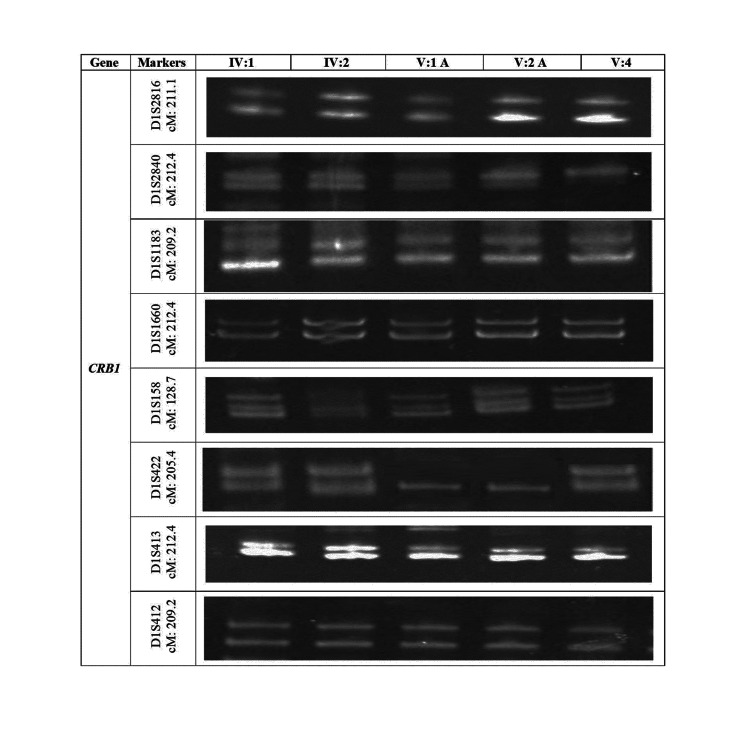
Electrophoresis of the RP01 family, marked with ethidium bromide in a nondenaturing polyacrylamide gel at a concentration of 8%.

DNA samples (IV:1, IV:2, V:1, V:2, and V:3), comprising three non-affected and two affected members of the family, were genotyped. No linkage was established using microsatellite markers for other selected genes (RP1, TULP1, RPE65, ABCA4). However, linkage was found using the microsatellite marker DIS422 for gene CRB1, and all non-affected members showed a heterozygous pattern for the mentioned marker (Figure 3).

**Figure 4:**
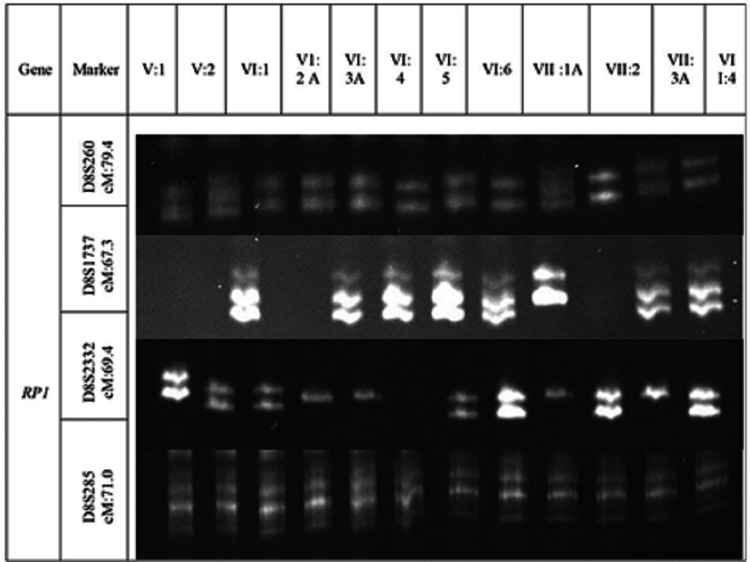
Electrophoresis of the RP02 family, marked with ethidium bromide in a nondenaturing polyacrylamide gel at a concentration of 8%.

DNA samples of 12 individuals of family (IV:1, IV:2, VI:1, VI:2, VI:3, VI:4, VI:5, VI:6, VII:1, VII:2, VII:3 and VII:4) containing of eight non-affected and four affected members were analyzed and genotyped. No linkage was established using microsatellite markers for other selected genes (CRB1, TULP1, RPE65, ABCA4). However, linkage was found using the microsatellite marker D8S2332 for gene RP1, and all non-affected members were heterozygous for D8S2332 (Figure 4).

**Figure 5:**
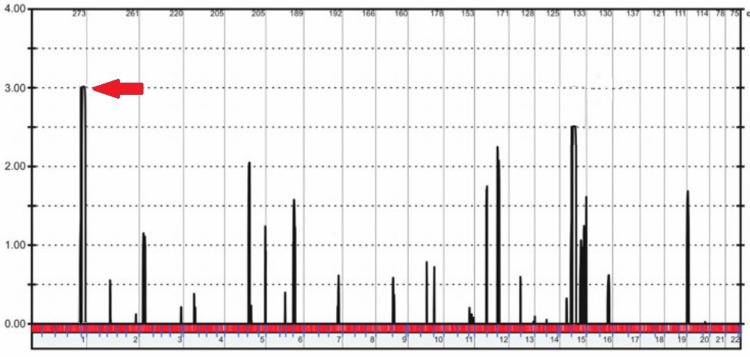
You can see the outcomes of the linkage analysis performed on the Illumina 6K arrays for RP01. Using Easy Linkage, we were able to locate the highest LOD-score summits. Red lines point to the peaks that are located in the same areas as the genes that were found to be mutated.

**Figure 6:**
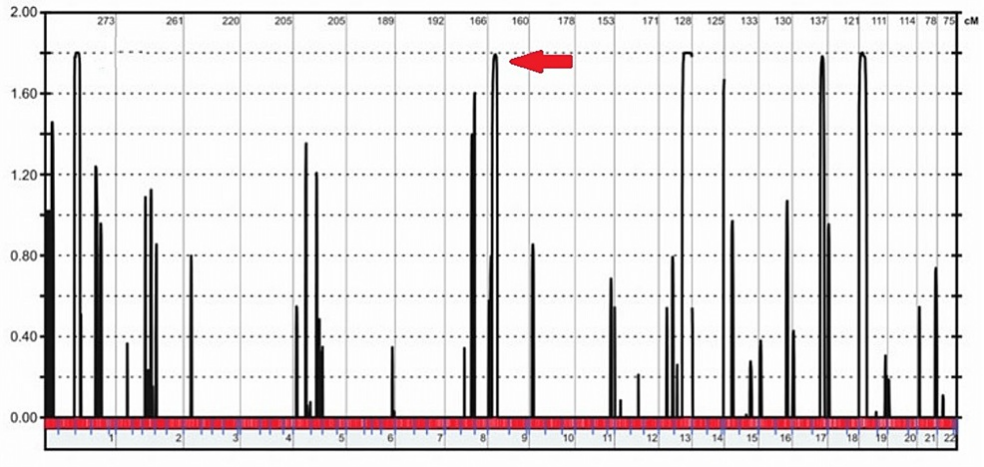
The figure shows the outcomes of a linkage study performed on Illumina 6K arrays for RP02. Using EasyLinkage, we were able to locate the greatest LOD-score summits. Red lines point to the peaks that correlate to the areas containing the genes in which changes were found.

The Illumina 6K array and multipoint linkage analysis were used to search for potential chromosomal areas containing the genetic flaw in two consanguineous households with impacted members. Only one peak on chromosome 1 was found in the RP01 family, and its highest multipoint LOD value was 3.00 (Figure 5). Multiple peaks on chromosome 8 with multipoint LOD values of 1.80 were found to be at risk for carrying the genetic abnormality in family RP02 (Figure 6).

The crumbs homolog 1 (CRB1) gene was found on chromosome 1 and was shared by two afflicted siblings in the RP01 family. The mutant study of CRB1 revealed a glycine for a valine (c.1152T>G; p.V243G) substitution in a homozygous state. In family RP02, however, all four afflicted members shared only three copies of the big homozygous area on chromosome 8 that contains the RP1 gene, and sequencing showed a cytosine to thymine shift that causes a proline to leucine mutation (c.3419C>T; p. P1035L) (Table 2).

**Table 3:** Identification of homozygous regions and mutations

Family	Affected Individuals	SNP array	Genomic position	Size (Mb)	arRP gene in the region	Mutation (DNA)	Predicted effect (protein)
RP01	02	III 6k	chr 1: 187.031.387–209.381.921	21.3	CRB1	c.1152T>G	p.V243G
RP02	04	III 6k	chr8:54,509,422-54,871,720	19.3	RP1	c.3419C>T	p. P1035L

## Discussion

RP is a heterogeneous group of hereditary retinopathies with a large number of known mutations [11], several disease-causing genes, and extremely diverse clinical outcomes [7,9]. In the past two decades, remarkable progress has been achieved in locating the genes responsible for hereditary retinal conditions, including RP. The link between genes, mutations, and clinical data has unavoidably grown quite complicated [12]. Greater knowledge of the biological foundation of vision and insights into the mechanisms involved in retinal pathology are just a couple of the numerous effects that might result from successfully identifying the origins of hereditary retinal illnesses [13-15]. Finding the genes and mutations linked with RP requires both gene discovery and mutation screening in affected people and their families. Our aim was to identify the gene mutation that causes RP in Pakistani families using homozygosity mapping, which will advance the search for remedies.

Studies have identified several genes associated with RP in Pakistani families, including RP1, RP2, RP9, DHX38, and PRPF31 [9,16,17]. Mutations in these genes account for a significant proportion of cases of autosomal recessive RP in Pakistan. Ali et al. [9] found linkage analysis, but most of these families contain compound heterozygous variants in either a known arRP gene or an as-yet-undiscovered arRP gene [13,15,16]. To identify chromosomal areas that might house the underlying genetic flaw, homozygosity mapping and mutation analysis were conducted in this research.

In our study, all members of the RP02 family who were not impacted by the linkage were heterozygous, whereas those who were affected were homozygous, according to the microsatellite marker D8S2332, which was used to identify the linkage in the RP1 gene. Additionally, the CRB1 gene linkage in the RP01 family was found using the microsatellite marker DIS422, and all unaffected people showed a heterozygous pattern for this marker, while afflicted members were homozygous. In the study by Umm-e-Aiman et al. [18] on the autosomal recessive RP family RP01, there was one dead person and three afflicted members. In the autosomal recessive RP family RP02, there was one dead person and one afflicted person. Due to homozygosity for the genes ZNF513, TULP1, RP1, and MERTK in both affected and unaffected members of the two RP families, no linkage was detected in these families. However, few pieces of research had linked these genes to RP families [19,20].

The genetic flaw was sought by performing a multipoint linkage analysis to locate possible hotspots in the genome. A single peak on chromosome 1 was found in the RP01 family, with the highest multipoint LOD value of 3.00. Multiple points on chromosome 8 were found to have multipoint LOD values of 1.80, suggesting that they may be the site of the genetic abnormality in family RP02. Leucine was replaced for a proline residue as a consequence of the cytosine to thymine transition in the RP1 gene analysis (c.3419C>T; p. P1035L), and glycine was used in place of valine in the CRB1 mutation research (c.1152T>G; p.V243G). In this study by Latif et al. [21], 11 members of a large consanguineous family were selected from Azad Jammu and Kashmir, where they underwent linkage mapping analysis and Sanger sequencing confirmation. This family's non-syndromic autosomal recessive RP was caused by homozygous c.2536G>A mutations in the CRB1 gene.

The RP genetics of Pakistani families are complex and entail numerous gene mutations. These mutations are essential for genetic counseling and targeted treatments for this dreadful disease. We provide additional information regarding the phenotypic and mutational spectrum of this variant of RP. These findings help explain the molecular genetic factors underlying inheritable RP in Pakistan, which impact genetic counseling, detection, prognosis, and possibly the selection of patients eligible for genetic therapies to halt its progression.

Limitation

This research was confined to families with retinitis pigmentosa patients. Due to a shortage of resources, only five genes (*CRB1, RP1, TULP1, RPE65,* and *ABCA4*) were randomly selected. 

## Conclusions

In conclusion, our research supports the efficacy of homozygosity mapping, which is accurate and thorough while being reasonably priced, for locating pathogenic mutations in consanguineous families with arRP. This method is important for providing accurate clinical diagnoses and genetic counseling in remote parts of Pakistan while also promoting public knowledge of arRP and the dangers of consanguineous marriage.
